# Editorial: The application of artificial intelligence in interventional neuroradiology

**DOI:** 10.3389/fneur.2022.1112624

**Published:** 2023-01-05

**Authors:** Yuhua Jiang, Jian Lv, Youxiang Li, Yanling Zhang

**Affiliations:** ^1^Department of Neurosurgery, Beijing Neurosurgical Institute and Beijing Tiantan Hospital, Capital Medical University, Beijing, China; ^2^Department of Neurointerventional Engineering and Technology, Beijing Engineering Research Center, Beijing, China; ^3^Department of Radiology, Beijing Tiantan Hospital, Capital Medical University, Beijing, China

**Keywords:** interventional neuroradiology, artificial intelligence, cerebrovascular disease, cerebrovascular interventional therapy, prediction models

## Introduction

As editors of this Research Topic, it was our pleasure to introduce novel findings and new achievements in the application of artificial intelligence (AI) in interventional neuroradiology. Cerebrovascular disease is becoming an increasingly important cause of death and intervention therapy has become the mainstay treatment for this disease. However, there is a need to assess the efficacy and safety of endovascular therapy. In recent years, AI technology has advanced rapidly and has shown great promise in solving complicated problems. It also possesses strong potential to improve the clinical application of cerebrovascular interventional therapy.

This Research Topic consists of 10 papers, including seven original research articles, one system review, one mini review, and one commentary. The purpose of this Editorial is to summarize the key findings and perspectives presented in each of the accepted articles.

The basic work of applying the AI technology in practice is to design and establish a database. So, You et al. introduced a protocol for constructing a multicenter database based on CTA images of IAs. This protocol described how to collect research data, conduct aneurysm segmentation, and annotation. This study exemplifies the paradigm of building a big database for artificial intelligence.

A similar study has been performed previously (1). From such studies we know that a well-established database of the AI model can directly improve the performance of the model.

Compared to statistical regression models, machine learning (ML) models, which are part of AI technology, can identify non-intuitive patterns in variables which might be missed by statistical tests. Endovascular treatment strategies can be optimized by employing ML models to predict therapeutic outcomes.

However, clinicians lack the skills to handle ML data scientific, which hinder the development of such models. Ou et al. designed three models for predicting IAs endovascular treatment outcome. They created an automated machine learning (AutoML)-derived model which showed better performance compared with the manually trained ML and statistical models. The AutoML model has the advantage of simplifying the process of building a ML model without relying on experts. Therefore, it can be used by people without expertise in artificial intelligence. However, these studies had several limitations. For instance, they did not assess the calibration of various algorithms as mentioned in the commentary by Huber et al.

Assessment of clinical outcomes is essential for acute ischemic stroke (AIS) patients. Jabal et al. built ML models for pre-intervention prediction of the 90-day dichotomized modified Rankin Scale (mRS-90) scores for AIS patients who underwent thrombectomy, using clinical and radiological information extracted from CTA and CTA with the e-stroke software. The authors used various ML algorithms including *k*-nearest neighbors, random forests (RF), gradient boosting (GB), and Extreme Gradient Boosting (XGBoost) and found that XGBoost was the best performing classifier.

Although endovascular treatment has become the mainstay treatment for IAs, its related complications should not be ignored. Clinicians should balance between the risk of complications from endovascular treatment and the risk of IAs rupture. Tian et al. constructed several ML prediction models to study endovascular procedure-related complications of IAs and found that the ANN models had the best performance. In the study, 443 patients were enrolled, and the three most significant features were distal aneurysm, aneurysm size, and treatment modality as determined by the Shapely Additive explain (SHAP) and feature importance analysis.

Pipeline embolization device (PED) is the most commonly used flow diverter service for the treatment of IAs. In-stent stenosis (ISS) is a common complication after PED placement and might adversely affect long-term prognosis. Wei et al. built ML prediction models using clinical, laboratory, and imaging data obtained from 435 patients. They compared the prediction performance of five ML algorithms including elastic net (ENT), SVM, XGBoost, Gaussian Naïve bayes, and random forest. Through SHAP analysis, they found that internal carotid artery location was the most important predictor.

Notably, AI prediction models cannot override traditional statistical models totally because of the data and technology limitations. The continuous advancement of AI and medical image processing technology, is expected to provide multi-dimensional information upon which precise AI prediction models will be built. Similar opinions were held by Zeng et al. In their systematic review, they enrolled 16 articles, including 19 ML and DL models for predicting prognosis of stroke patients with large vessel occlusion (LVO). They found that AI did not show an overall advantage over existing prognostic scores. Therefore, whether ML and DL methods can improve prediction of stroke outcomes in LVO still need to be further clarified. Marasini et al. reviewed AI methods applied in IAs detection, IAs screening, IAs rupture prediction, IAs clinical decision support and workflow enhancement of IAs. They reported that AI can handle large numbers of variables and identify non-linear relationships among them. However, despite significant advances in the field of AI, its application in many areas, particularly in non-imaging data is at the foundational stage. Therefore, further studies are needed to improve the prediction performance of AI for periprocedural complications related to endovascular therapy.

## Author contributions

YJ and JL wrote the original manuscript. YL and YZ reviewed and edited the manuscript. All authors contributed to the article and approved the submitted version.
